# Hydrogen induced interface engineering in Fe_2_O_3_–TiO_2_ heterostructures for efficient charge separation for solar-driven water oxidation in photoelectrochemical cells†

**DOI:** 10.1039/d0ra09655e

**Published:** 2021-01-21

**Authors:** Aadesh P. Singh, Richard Baochang Wang, Camilla Tossi, Ilkka Tittonen, Björn Wickman, Anders Hellman

**Affiliations:** Department of Electronics and Nanoengineering, School of Electrical Engineering, Aalto University P.O. Box 13500 00076 Aalto Finland aadesh.singh@aalto.fi; Division of Chemical Physics, Department of Physics, Chalmers University of Technology SE-412 96 Göteborg Sweden anders.hellman@chalmers.se +46 31 772 5611

## Abstract

Semiconductor heterostructure junctions are known to improve the water oxidation performance in photoelectrochemical (PEC) cells. Depending on the semiconductor materials involved, different kinds of junctions can appear, for instance, type II band alignment where the conduction and valence bands of the semiconductor materials are staggered with respect to each other. This band alignment allows for a charge separation of the photogenerated electron–hole pairs, where the holes will go from low-to-high valance band levels and *vice versa* for the electrons. For this reason, interface engineering has attracted intensive attention in recent years. In this work, a simplified model of the Fe_2_O_3_–TiO_2_ heterostructure was investigated *via* first-principles calculations. The results show that Fe_2_O_3_–TiO_2_ produces a type I band alignment in the heterojunction, which is detrimental to the water oxidation reaction. However, the results also show that interstitial hydrogens are energetically allowed in TiO_2_ and that they introduce states above the valance band, which can assist in the transfer of holes through the TiO_2_ layer. In response, well-defined planar Fe_2_O_3_–TiO_2_ heterostructures were manufactured, and measurements confirm the formation of a type I band alignment in the case of Fe_2_O_3_–TiO_2_, with very low photocurrent density as a result. However, once TiO_2_ was subjected to hydrogen treatment, there was a nine times higher photocurrent density at 1.50 V *vs.* the reversible hydrogen electrode under 1 sun illumination as compared to the original heterostructured photoanode. *Via* optical absorption, XPS analysis, and (photo)electrochemical measurements, it is clear that hydrogen treated TiO_2_ results in a type II band alignment in the Fe_2_O_3_–H:TiO_2_ heterostructure. This work is an example of how hydrogen doping in TiO_2_ can tailor the band alignment in TiO_2_–Fe_2_O_3_ heterostructures. As such, it provides valuable insights for the further development of similar material combinations.

## Abstract 2

Approximate energy band diagram and charge transfer mechanism for the Fe_2_O_3_–TiO_2_ photoanode (left) and Fe_2_O_3_–H:TiO_2_ photoanode (right) with external applied anodic potential under illumination.

## Graphical abstract

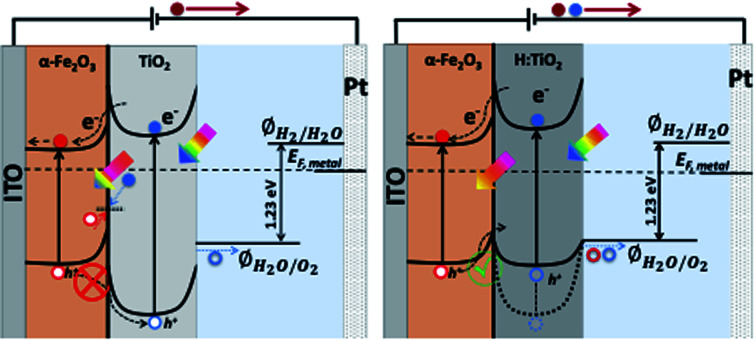

## 1. Introduction

Solar energy-assisted splitting of water into its constituents, hydrogen and oxygen, in a photoelectrochemical (PEC) cell, represents a promising route to convert solar energy into more useful chemical fuels.^1,2^ The water reduction process can produce hydrogen, where the required electrons can be generated *via* the water oxidation reaction at the surface of a photoanode through PEC reactions. However, the water oxidation reaction is a demanding electrochemical process, requiring an oxidatively robust and yet inexpensive semiconducting material as the photoanode.^3^ Despite tremendous efforts, developing a highly active photoanode for water oxidation at low cost remains a significant challenge. After the first report on water splitting by Fujishima and Honda,^4^ titanium dioxide (TiO_2_) in its anatase phase has been extensively studied in solar-driven photocatalytic processes.^5–7^ However, for practical applications, the large bandgap of TiO_2_ (∼3.2 eV) requires solar radiation with wavelength below 388 nm to create an electron–hole pair; thereby, limiting the overall efficiency of TiO_2_ when illuminated under real Sun conditions.^8,9^ Another material of high interest for PEC water splitting is hematite (α-Fe_2_O_3_), due to its suitable band gap (2.1 eV), high stability over a wide range of pH and potentials, and low material cost.^10,11^ In spite of these various advantages, the solar-to-hydrogen conversion efficiency of α-Fe_2_O_3_ falls well below the theoretical maximum value (≈12.9%) due to a number of factors such as (i) bulk charge recombination, (ii) interfacial carrier trapping and recombination, (iii) surface trapping and recombination, and (iv) improper band positions for unassisted water splitting^12^ (see Fig. S1† for further information).

**Fig. 1:**
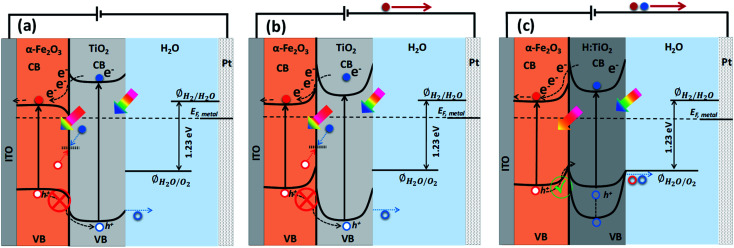
Approximate energy band diagram and charge transfer mechanism of (a) Fe_2_O_3_–TiO_2_ photoanode without external applied anodic potential, (b) Fe_2_O_3_–TiO_2_ photoanode with external applied anodic potential and (c) Fe_2_O_3_–H:TiO_2_ photoanode with external applied anodic potential.

To address the issues with solely α-Fe_2_O_3_ and TiO_2_ based oxide semiconductors and design a photoanode for PEC water oxidation with high solar-to-hydrogen conversion efficiency, the heterostructure of these two semiconductors has been recognized as an attractive candidate to enhance the photocurrents and lower the onset potential.^13,14^ The combined properties of α-Fe_2_O_3_ and TiO_2_ allow the heterostructures to absorb a wider range of photons, thanks to the relatively narrow band gap of α-Fe_2_O_3_.^15^ Also, the formation of heterostructures between α-Fe_2_O_3_ and TiO_2_ can allow band structure engineering to manipulate surface/interface properties for charge transfer/separation, thereby enhancing the water oxidation performance.^16^ In particular, the valence and conduction band alignment mechanisms at the interface are crucial for the separation of photogenerated charge carriers.^17^ In both oxide semiconductors, the Fermi levels depend on the concentrations of the conduction electrons and hence on the oxygen vacancy concentrations. By appropriate adjustments of the Fermi levels *via* defect concentration, the valence band edge in the bulk of the semiconductor may be brought to a common equilibrium. Using the available data^18^ of the electron affinities for Fe_2_O_3_ (4.71 eV) and TiO_2_ (4.33 eV), together with the generally accepted model of heterojunctions, the discontinuity at the conduction bands (CB) is estimated to be 0.38 eV while that for the valence bands (VB) is estimated to be 0.42 eV. Owing to the mutual positions of Fe_2_O_3_ and TiO_2_ conduction band edges, photogenerated electrons in TiO_2_ can be easily transferred to Fe_2_O_3_ and injected into the Indium Tin Oxide (ITO) substrate, and subsequently can migrate through the external electric circuit to reduce water at the cathode, thus suppressing detrimental recombination effects (Fig. 1a). On the other hand, the VB offset at the interface by 0.42 eV act as an energy barrier that blocks the hole transfer from the α-Fe_2_O_3_ to the TiO_2_ layer and prevents the water oxidation reaction on the surface of TiO_2_ (Fig. 1a). This means the heterostructure between α-Fe_2_O_3_ and TiO_2_ forms a type I band alignment at the semiconductors' interface, and the hole transport from α-Fe_2_O_3_ to TiO_2_ is energetically impeded, as depicted in Fig. 1a and b.

However, in the case of a thin pours TiO_2_ layer (<100 nm) over Fe_2_O_3_ and at applied anodic potential, the depletion region and upward band bending in TiO_2_ at Fe_2_O_3_–TiO_2_ nano-heterostructure in contact with the electrolyte may extend into the α-Fe_2_O_3_. This high band bending in the thin TiO_2_ overlayer, under the influence of anodic potential, can provide a channel to transfer the photogenerated holes from Fe_2_O_3_ to TiO_2_ where they participate in the water oxidation reaction, thereby improving the water oxidation performance of the Fe_2_O_3_–TiO_2_ photoanode. Barreca *et al.*^15^ reported that the use of external potential could transfer of photogenerated holes from porous hematite to TiO_2_ layers that can improve the PEC response under simulated light. However, in the case of thick and dense TiO_2_ layer over α-Fe_2_O_3_, the mismatched band alignment can hinder the charge transfer process at Fe_2_O_3_–TiO_2_ heterostructure, thereby constitute the limiting factor in PEC applications. Therefore, for swift transfer of photogenerated holes from VB of α-Fe_2_O_3_ to the VB of TiO_2_, band edge and band gap tailoring in TiO_2_ are required to form a type II band alignment at the Fe_2_O_3_–TiO_2_ heterostructure interface. In our previous study,^8,9,17^ we demonstrated that a high temperature hydrogen treatment in TiO_2_ under partial pressure can modify the structural, optical, and electrical properties and significantly improve the photocatalytic and photoelectrochemical performance. An upshift of the valence band of TiO_2_ was achieved after the high temperature hydrogen treatment at partial pressure that was useful for band edge engineering at TiO_2_–BiVO_4_ heterostructure. However, a downshift of valance band maximum (VBM) was also obtained when TiO_2_ thin films were grown *in situ* in hydrogen plasma.^19^

In this study, first-principles calculations on a model Fe_2_O_3_–TiO_2_ heterostructure show that the strain at the interface shifts the valance and conduction band positions and affects the bandgap of the TiO_2_ layer. Furthermore, the calculations show that neutral and positive charged H interstitials are energetically preferred under hydrogen treatment conditions. These interstitials introduce states in the bandgap of TiO_2_, which can assist in the transfer of holes through the TiO_2_ layer. To challenge the theoretical predictions, well-defined planar Fe_2_O_3_–TiO_2_ heterostructures were manufactured. The experimental results show that Fe_2_O_3_–TiO_2_ initially forms a type I band alignment, prohibiting the transfer of holes through TiO_2_. However, after hydrogen treatment, the Fe_2_O_3_–H:TiO_2_ heterostructure seems to form a type II band alignment; thus, hole transfer becomes possible, leading to an enhanced PEC response.

## 2. Experimental section

### 2.1 Preparation of α-Fe_2_O_3_ thin films

The ultra-thin hematite films were prepared onto indium-doped tin oxide (SnO_2_:In, ITO, PGO GmbH, sheet resistance < 20 Ω sq^−1^) substrate by using physical vapor deposition technique, followed by annealing of Fe coated ITO substrate in air atmosphere.^20^ Briefly, all the substrates were cleaned by using soap solution, acetone, and deionized water followed by ultra-sonication in isopropanol for 5 minutes and finally with oxygen plasma for two minutes. Before deposition, 1/3 area (5 mm × 10 mm) of the substrate was covered with a thermal tape for making the electrical contact later for PEC measurements. To fabricate the ultra-thin hematite film (approximately 25 nm), a 10 nm thick Fe films were deposited on ITO substrate by physical vapor deposition (PVD 225, Kurt J. Lesker, base pressure < 5 × 10^−7^ mbar) and annealed in air atmosphere at 350 °C for 8 h with a heating rate of 4 °C min^−1^. The thickness of the Fe film was measured *in situ* during deposition using a quartz-crystal microbalance monitor integrated in PVD system. After annealing, the samples were allowed to cool down to room temperature naturally.

### 2.2 Preparation of Fe_2_O_3_–TiO_2_ and Fe_2_O_3_–H:TiO_2_ heterostructures

Thin films of TiO_2_ were deposited onto ITO substrates and also on hematite coated ITO substrate by sol–gel technique.^6^ In short, a transparent gel solution of titanium dioxide was prepared by mixing 3 ml titanium tetra-isopropoxide (TTIP, 97% pure) in 20 ml ethanol in the presence of diethanolamine. The solution was stirred for 4 h at room temperature to enhance the reaction rate between diethanolamine and TTIP, and finally, it was converted into a gel. This gel solution was applied on α-Fe_2_O_3_/ITO substrate and uniformly coated with the help of a spin coating unit at 3000 rpm for 1 minute. A thin layer of TiO_2_ was achieved by the deposition of prepared gel over the α-Fe_2_O_3_/ITO substrates. After deposition, the prepared TiO_2_–Fe_2_O_3_ heterostructure was allowed to dry for 10 min at 80 °C and further annealed at 350 °C for 4 h. The hydrogen treatment was carried out by annealing the Fe_2_O_3_–TiO_2_ heterostructures at 300 °C in 4% H_2_ in Ar at atmospheric pressure for 6 h. We also prepared a hydrogen doped TiO_2_ thin film photoanode under the same annealing conditions to see the effect of hydrogen doping individually on optical, electrical, and PEC properties.

### 2.3 Material characterization

The chemical phase of the prepared samples was determined by using a confocal Raman microscope (alpha300 R; WITec) with a 488 nm laser pulse as an excitation source. The surface morphology of the bare and TiO_2_ coated α-Fe_2_O_3_ samples was examined by field emission scanning electron microscope (FE-SEM) using a Zeiss Supra 60 VP microscope operated at an acceleration voltage of 10 kV. The optical absorption of all the samples was measured with the help of a Cary 5000 spectrophotometer (Varian). X-ray photoelectron spectroscopy (XPS) spectra were acquired in a PerkinElmer Phi 5500 setup (base pressure < 10^−10^ mbar) using AlK_α_ radiation of 1.4866 keV. The XPS spectra were shifted using the Fe(2p_3/2_) peak corresponding to 710.9 eV as a reference.

### 2.4 Photoelectrochemical measurements

For electrochemical measurements, thin films of α-Fe_2_O_3_, TiO_2_, H:TiO_2_, Fe_2_O_3_–TiO_2_ and Fe_2_O_3_–H:TiO_2_ heterojunctions were converted into the photoelectrodes with an active surface area of about 0.50 cm^2^. All the (photo)electrochemical measurements, current–voltage (*I*–*V*), capacitance–voltage (*C*–*V*), and electrochemical impedance spectroscopy (EIS), were conducted in a three-electrode configuration using a H-type PEC cell made of glass and fitted with a flat optical quartz window containing 0.1 M NaOH as electrolyte (pH = 12.9). The PEC cell was controlled by using the Gamry Ref. 600 potentiostat and a solar simulator (SKU SS150, Sciencetech Inc.) with an output intensity of 100 mW cm^−2^ as an illumination source. Here, the prepared photoelectrodes were used as a working electrode, Pt wire as a counter electrode, and Ag/AgCl as a reference electrode. For better representation of our results, the Ag/AgCl reference potential was converted into the reversible hydrogen electrode (RHE) potential by using the following formula: *V*_RHE_ = *V*_Ag/AgCl_ + 0.059pH + *V*^o^_Ag/AgCl_, where *V*^o^_Ag/AgCl_ is the standard potential of Ag/AgCl at 25 °C (0.1976 V *vs.* the standard hydrogen electrode, SHE). The cyclic voltammetry sweep scans in the potential range between 0 and 2.0 V_RHE_ at a scan rate of 10 mV s^−1^ were performed to obtain the *I*–*V* characteristics under dark and illumination. The photocurrent density (*J*_ph_) has been calculated by subtracting the dark current from the current measured under illumination and devised by the geometrical area of the photoanode. Electrochemical impedance spectroscopic (EIS) measurements under illumination were carried out in the frequency range 10^5^ and 0.1 Hz at applied potentials between 1.23 V_RHE_. Nyquist plots obtained under illumination were fitted using the software EIS spectrum analyzer. Mott–Schottky analysis was performed at an applied frequency of 1 kHz in the dark condition in a potential window between 0 and 1.5 V_RHE_. The obtained Mott–Schottky curves (1/*C*^2^*versus V*_RHE_) were used to determine the donor density (*N*_D_) and flat band potential (*V*_fb_) by using the Mott–Schottky equation: *C*^−2^ = (2/*qε*_o_*ε*_s_*N*_D_)[*V* − *V*_fb_ − *kT*/*q*], where *ε*_o_ is the permittivity of the vacuum, *ε*_s_ is the dielectric constant of the hematite, *q* is the electronic charge, and *kT*/*q* is the thermal voltage (26 meV at room temperature). The donor density was calculated using the equation, *N*_D_ = 2/(*ε*_o_*ε*_s_*q*)[d(1/*C*^2^)/d*V*]^−1^, from the slope of the linear region, between 0.6 and 1.0 V_RHE_, of Mott–Schottky plots.

### 2.5 Computational methods

The first-principles calculations were performed using density functional theory (DFT) as implemented in the VASP package.^21–23^ The interaction between the valance electrons and the core follows the projector augmented wave (PAW) method.^24^ PAW potentials with the valence states 1s for H, 2s and 2p for O, 3d and 4s for Fe, and 3d and 4s for Ti have been employed. A plane wave basis with a kinetic energy cut-off 700 eV was used. To improve convergence, a Gaussian smearing broadening of the Fermi surface of 0.1 eV was employed. The exchange–correlation (XC) interaction was treated at the level of the generalized gradient approximation (GGA) using the XC-functional of Perdew, Burke, and Ernzerhof (PBE).^25^ In the DFT+U calculations, the rotationally-invariant scheme proposed by Dudarev *et al.*^26^ and a *U* − *J* = 4.3 eV on Fe atom and *U* − *J* = 5.2 eV on Ti atom are employed in all our calculations.^27,28^ The HSE06 method, as implemented in VASP, was employed to study H defects in anatase TiO_2_.^29,30^ A kinetic cut-off of 600 eV was applied in all calculations. The interface system is composed of a six-layer thick Fe_2_O_3_ with a (0001) termination of p(2 × 2), that is joined with a four-layer thick anatase TiO_2_ with a (101) termination of p(1 × 2). Owing to the lattice mismatch between Fe_2_O_3_ and TiO_2_, the interface will introduce strain to the system. Here the lattice cell (*a* = *b* = 10.17 Å, *a* = 120°) of Fe_2_O_3_ (0001) surface is kept fixed, which implies a strain to the original lattice cell (*a* = 11.35 Å, *b* = 10.23 Å, alpha = 111.69°) of the TiO_2_ (101) surface. The strain amounts to 10% compression strain on the *a*-axis and 0.6% compression strain to the *b*-axis, and an increase of the angle by 7.4%. During the relaxation of the interface system, the anatase TiO_2_ reconstructs to amorphous TiO_2_ (a-TiO_2_). Here, the term amorphous is used to indicate that the reconstructed TiO_2_ in the interface model can not be identified with any known phase (rutile, anatase, brookite, *etc.*) of TiO_2_. However, it should be noted that due to the periodic conditions, this phase should not be considered as genuinely amorphous TiO_2_. Defect calculations were carried out in a 
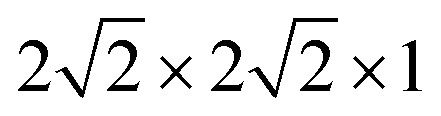
 (96 atoms) supercell of anatase TiO_2_, with a *Γ*-centered 1 × 1 × 1 *k*-point sampling. The effect of higher concentrations of defects is modeled by a 
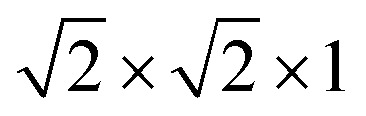
 (24 atoms) supercell and a 1 × 1 × 1 (12 atoms) unit cell, using a 3 × 3 × 1 Monkhorst–Pack (MP) sampling.^31^ All geometries were relaxed until the maximum force was less than 0.05 eV Å^−1^. The relative stability of the various defects in charged and/or neutral states is determined by the formation energy,

1 Δ*E*_f_ = *E*_t_(D^*q*^) − *E*_t_(TiO_2_) − Δ*n*_O_*μ*_O_ − Δ*n*_H_*μ*_H_ + *qE*_F_ + *E*_corr_,

where *E*_t_(D^*q*^) is the total energy of the supercell containing a defect D in charge state *q* and *E*_t_(TiO_2_) is the total energy of a perfect crystal in the same supercell. Δ*n*_O_ and Δ*n*_H_ are the number of O atom and H atom changed in the defective supercell with respect to the perfect one. *μ*_O_ and *μ*_H_ are the chemical potential of O atom and H atom. *E*_F_ and *E*_corr_ refer to the energy of the Fermi level and the Madelung corrections for the electrostatic interaction between the homogeneous background charge and charged defects. The chemical potential of the O atom is selected to satisfy the stability condition of TiO_2_. In the extreme O-rich limit, the *μ*_O_ is set to 
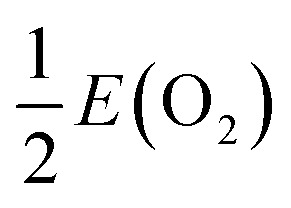
, where *E*(O_2_) is the calculated total energy of isolated O_2_ molecule. In the extreme O-poor limit, *μ*_O_ corresponds to the formation of Ti_2_O_3_, which has a relationship of *μ*_O_(O-poor) = *μ*_O_(O-rich) − 3.97 eV calculated by HSE06. *μ*_H_ is selected as one half of the total energy of isolated H_2_ molecule, ½*E*(H_2_).

## 3. Results

### 3.1 Simulation results

**Fig. 2:**
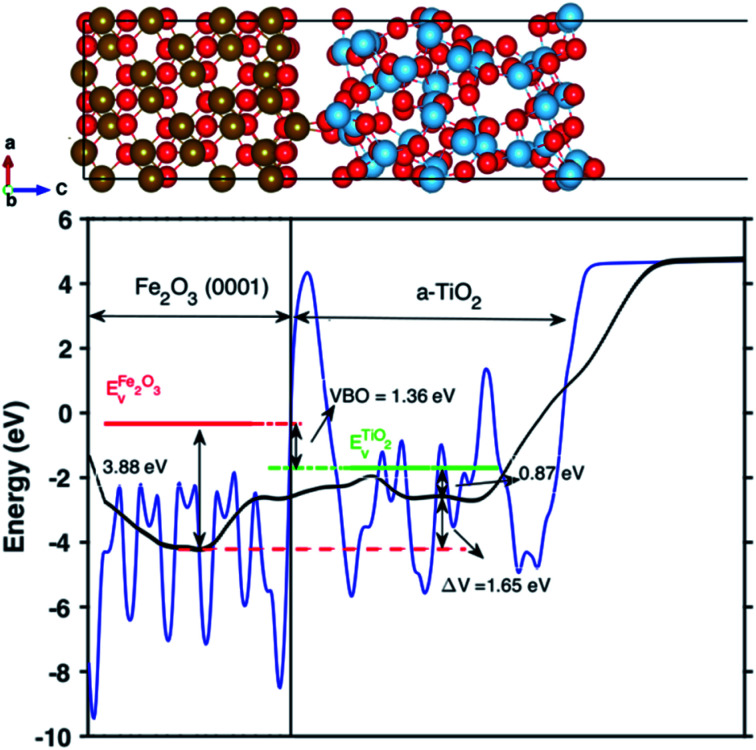
Up panel is the relaxed structures from the PEB + U calculation for the Fe_2_O_3_ and amorphous TiO_2_ interface. Bottom panel is the calculated valence band offset (VBO) of the interface with PBE+U methods. The black line is the calculated macroscopically averaged electrostatic potential and the blue line is the electrostatic potential at the interface averaged along the thickness (*c*-axis) of the interface. The red line is the position of VBM of Fe_2_O_3_ and the green line is the position of VBM of bulk TiO_2_ with respect to vacuum level. The electrostatic potential across the interface is Δ*V* = 1.65 eV and the calculated VBO at the interface is 1.36 eV. The VBO of the joint interface is slightly larger than that of separated systems by 0.18 eV that indicating the creation of an interface dipole or double layers.^37^

The valence band offset (VBO) at the interface of Fe_2_O_3_ and a-TiO_2_ was calculated through the reference potential method originally introduced by Kleinman^32,33^ where the reference potential use the macroscopically averaged electrostatic potential.^34^ The calculated results are shown in Fig. 2. The VBO for the individual system, Fe_2_O_3_ (0001) and a-TiO_2_ thin film, were also calculated and are shown in the ESI (Fig. S2).† The blue curve in Fig. 2 refers to the electrostatic potential calculated using PBE+U method. The valence band edges of Fe_2_O_3_ and a-TiO_2_ were determined with respect to the macroscopically averaged electrostatic by performing calculations on the separated systems. The values of Fermi energy were calculated to 3.88 eV and 0.87 eV for Fe_2_O_3_ (0001) and a-TiO_2_ thin film, respectively. The macroscopically averaged electrostatic potential was calculated by,2
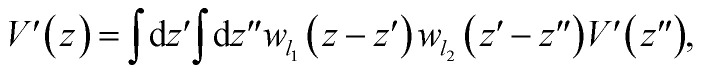
where 
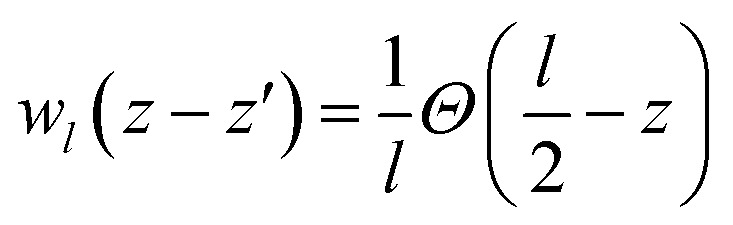
, *Θ* is the unit-step function, *l*_1_ and *l*_2_ are in the order of the (strained) thickness of hematite (0001) and amorphous TiO_2_ thin film along *z*, respectively.3
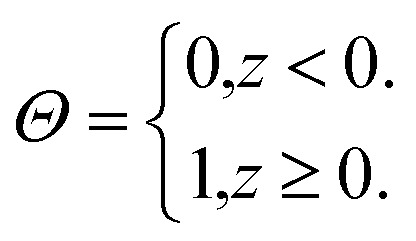
where *V*′(*z*) is the *xy*-plane averaged electrostatic potential and *d*_1_ and *d*_2_ are the inter-planar distances along the *z* direction (normal to the interface).^35,36^

The calculated *V*′(*z*) is the black line in Fig. 2. The VBO was calculated *via*,

4 VBO = Δ*E*_v_ + Δ*V*.

The calculated VBO for the individual system is −1.18 eV using DFT+U, which agrees well with the experimental observations (∼0.5 eV).^38^ However, the calculated VBO at the interface is around 1.36 eV, with the valence band edge of TiO_2_ sitting below the Fe_2_O_3_, which is 0.18 eV higher than the separated systems indicating an interface dipole or double layer were created at the interface. The electrostatic potential across the interface is Δ*V* = 1.65 eV.

**Fig. 3:**
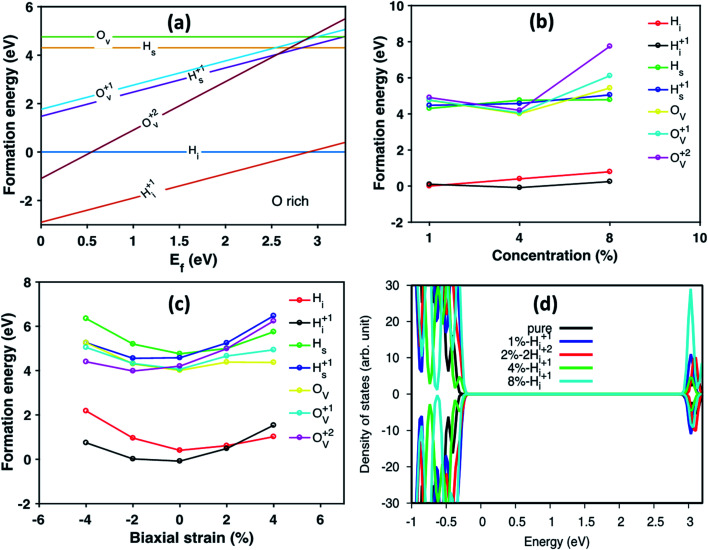
(a) The formation energy of different charged defects in anatase TiO_2_ under oxygen rich conditions corresponding to the current experiment condition. The Madelung corrections, *E*_corr_ is taken to be 0.1 eV in all our calculations. The *x*-axis is the Fermi energy level of anatase TiO_2_. O_v_ denotes the oxygen vacancy and can neutral (O_v_), +1 positively charged (O^1+^_v_) or +2 positively charged (O^2+^_v_). H_i_ is the interstitial H defect and H_s_ is the substitutional hydrogen defect. (b) The calculated formation energy of various defects in anatase varying with the defect concentration under O-rich condition. The Fermi energy is set to *E*_f_ = 3.0 eV. (c) The calculated formation energy of different charged/neutral defects under biaxial strain from −4% to +4% under O-rich condition. The Fermi energy is set to *E*_f_ = 3.0 eV. (d) The calculated density of states (DOS) of pure anatase and anatase with H_i_^+^ in different concentrations. The energy was aligned by the 1s state of O atom far away from the defect.

We further studied H defects and O vacancy in anatase TiO_2_, including their formation energy and electronic structure. The formation energy is calculated using eqn (1), and the results are shown in Fig. 3a, which corresponds to the results from the modeled supercell of 96 atoms. It is clearly shown that the positively charged H interstitial is the most favorable (with negative formation energy) defect when the Fermi energy is located above the valence band edge and below 2.9 eV, while the H interstitial becomes stable when *E*_f_ is larger than 2.9 eV (see Fig. 3). The formation energy of H interstitial is 0.04 eV. In contrast, O vacancies and substitutional H are less energetically favorable.

Next, we have studied the formation energies of different concentrations of defects in anatase TiO_2_ using different supercells, and the results are shown in Fig. 3b. Here, the formation energy plot is shown for O-rich conditions and the Fermi energy is set to 3.0 eV. As seen from Fig. 3b, the two most stable defect configurations are neutral and positively charged H interstitials, H_i_, where the positively charged H interstitial is the most favorable defect at high concentrations. The neutral H interstitial is the second state configuration when H concentration larger than 4 at%. The other defects are less stable as their formation energies are very high (larger than 3 eV). At high concentration (8 at%), the substitutional H_s_ becomes more stable than O vacancies. Interestingly, under O-poor conditions, the H_s_ is as stable as the H_i_ (the formation energy plot is shown in Fig. S3†).

When constructing the interface, a strain is induced automatically due to the lattice mismatch. Therefore, we also studied the stain effects on defect configurations for defect concentration of 4 at%. The calculated formation energy of different defects under biaxial strain is shown in Fig. 3c. It is interesting to notice that under biaxial tensile strain, the stable defect configuration changes to H_i_. Under compressive strain, positively charged H_i_^+^ remains as the most stable defect configuration. As shown in Fig. 3c, when changing from compressive to tensile strain, the stable configurations of defects are changed from charged ones to neutral ones, *e.g.*, H_s_ becomes more stable than H_s_^+^ and O_v_ becomes more stable than O_v_^+^ and O_v_^2+^. Similarly, the formation energy of defects calculated under O-poor conditions is shown in Fig. S4.† At such conditions, the defect configuration is changing from O_v_^2+^ to O_v_ as the strain changes from compression to expansion. However, H_i_^+^ is still the preferable defect under ambient condition (Fig. S4†). The DOS of anatase with different concentration of H_i_^+^ compared with pure anatase are shown in Fig. 3d. The energy of doped systems is aligned by shifting 1s state of O atom far away from the defect to that of pure anatase. It can be seen that doping with H_i_^+^ shifts the valence band edge upwards (∼0.1 eV) without changing the position of the conduction band edge. More specifically, the change of the valence band edge can be explained as the hybridization of 2p states of O atom bonded with H_i_^+^ and Ti 3d nearby (see Fig. S5†).

**Fig. 4:**
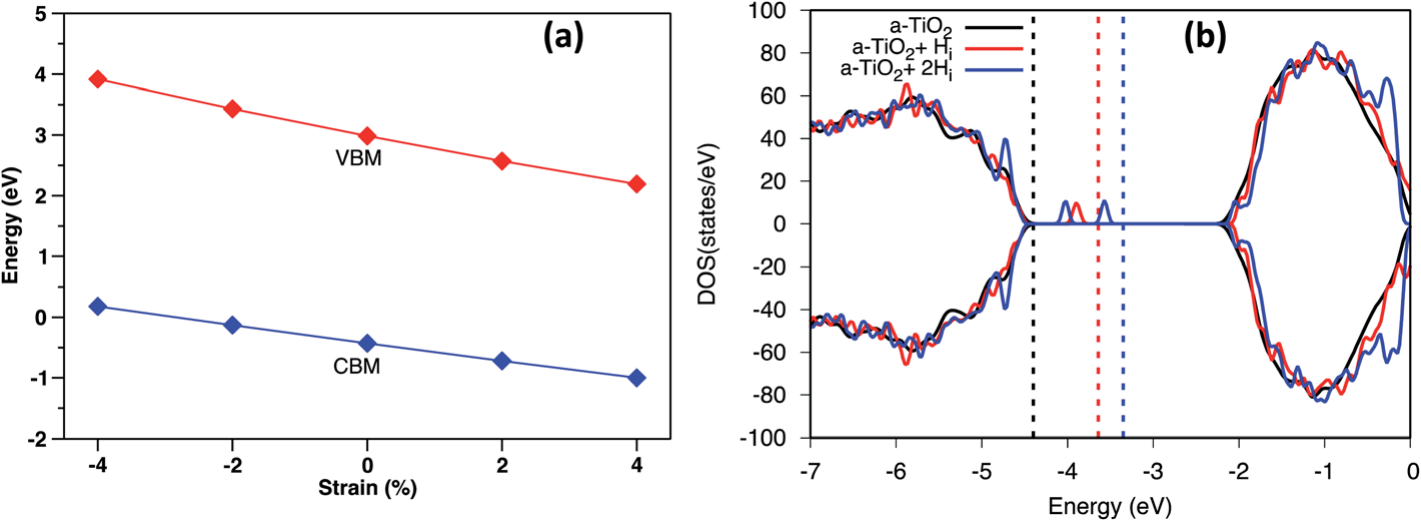
(a) The calculated VBM and CBM of anatase using HSE06 under different biaxial strain. (b) The DOS of H doped a-TiO_2_ compared with pure a-TiO_2_. The energy was aligned by the 1s state of O atom far away from the defect. The dashed line indicates the Fermi level of each system.

Under biaxial strain, due to lattice mismatch, the valence band maximum and conduction band minimum (CBM) of pure anatase (32 atoms supercell) under different strain are calculated and plotted in Fig. 4a. It is clear that both VBM and CBM decrease in energy as strain increases from compressive to tensile strain. When strain increases to 2%, the value of VBM decreases around 0.3 eV. Similarly, the CBM decreases around 0.4 eV. Therefore, the band gap is shrinking as strain increases. When doped with H_i_^+^, the VBM position is located below the VBM of pure anatase under 2% tensile strain, while the VBM remains unchanged with doped with H_i_ (shown in Fig. S4†). Hydrogen doping also introduces distortions to anatase TiO_2_, which results in the formation of the amorphous TiO_2_ at the interface. To shed further light on this, H doped amorphous TiO_2_ was studied with the PBE+U method, keeping the same parameters used for the interface. Doping with H_i_ is energetically favorable (shown in Fig. S4†) with a formation energy of −0.4 eV for single H_i_. The calculated DOS of H doped systems is shown in Fig. 4b. Compared with pure a-TiO_2_, single H_i_ introduces one occupied localized state on top of the valence band edge about 0.5 eV high in energy. The doping with H does not seem to change the averaged electrostatic potential, as can be seen from Fig. S5.† Adding two H_i_ introduce two localized states in the gap, but with slightly different energies. As more interstitial hydrogens are incorporated, we speculate that these states will eventually form energy levels in the range of up to ∼0.7 eV above the valence band edge. These energy levels work as the hole acceptance levels and help the transport of photo-excited holes generated from the Fe_2_O_3_ side to the H:TiO_2_ electrolyte side, where they can take part in the water oxidation reaction.

### 3.2 Experimental results

To test our hypothesis and to scrutinize the first-principles results experimentally, we have fabricated Fe_2_O_3_–TiO_2_ heterojunction electrodes in two steps. First, we deposited an ultra-thin α-Fe_2_O_3_ (25 nm) film on ITO substrate by thermal evaporation of Fe followed by air annealing at 350 °C. Further, the as-prepared α-Fe_2_O_3_ thin films were coated with TiO_2_ by sol–gel spin coating method followed by thermal annealing at 400 °C. In as-prepared Fe_2_O_3_–TiO_2_ heterostructure, the band edge engineering was achieved by hydrogen doping (Fe_2_O_3_–H:TiO_2_) by further annealing the Fe_2_O_3_–TiO_2_ heterostructure in 4% H_2_ in Ar at 300 °C at atmospheric pressure for 6 h.

To determine the crystal structures of α-Fe_2_O_3_, TiO_2_, and Fe_2_O_3_–TiO_2_ heterostructure thin films and possible phase changes after hydrogen doping in TiO_2_, Raman spectroscopy was performed, and its results are shown in Fig. S7a.† In pristine α-Fe_2_O_3_, normally seven phonon modes are expected in the Raman spectrum: namely two A_1g_ modes (225 and 498 cm^−1^) and five E_g_ modes (247, 293, 299, 412, and 613 cm^−1^), but the 293 and 299 cm^−1^ bands can only be resolved at temperatures ≤100 K. In the present case, six of the observed bands (A_1g_, 223 and 498 cm^−1^; E_g_, 244, 294, 410, and 607 cm^−1^) are detected, which agrees with the formation of the hematite phase (α-Fe_2_O_3_). On the other hand, the Raman spectra of both pristine and hydrogen doped TiO_2_ samples exhibit well-resolved TiO_2_ Raman peaks at 144 cm^−1^ (E_g_), 398 cm^−1^ (B_1g_), 515 cm^−1^ (E_g_), and 640 cm^−1^ (E_g_), indicating that anatase is the predominant species, except for 147 cm^−1^ (B_1g_), which is suppressed by a much stronger E_g_ peak at 144 cm^−1^. No phase change after hydrogen doping in TiO_2_ was observed (ESI Fig. S7b†). The Raman spectra of TiO_2_–Fe_2_O_3_ heterostructure shows the significant up-shift of E_g_ mode of TiO_2_ from 141 to 155 cm^−1^ and also other bands of TiO_2_. The shift of Raman bands by 14 cm^−1^ (inset of ESI Fig. S7b†) is probably due to the strain and defect states at the junction with α-Fe_2_O_3_ and lattice mismatch. Morphological characteristics were investigated by scanning electron microscopy (SEM) (ESI Fig. S8†) and transmission electron microscopy (TEM) (ESI Fig. S9†). SEM images of pristine α-Fe_2_O_3_ electrodes showed uniform deposition of a thin layer of a thickness of about 25 nm without any inter-particle pores, as shown in Fig. S8.† Optimized thickness of TiO_2_ layer (thickness < 10 nm) over the α-Fe_2_O_3_ layer on ITO clearly showed a smooth and dense coverage, as shown in Fig. S8c.†

**Fig. 5:**
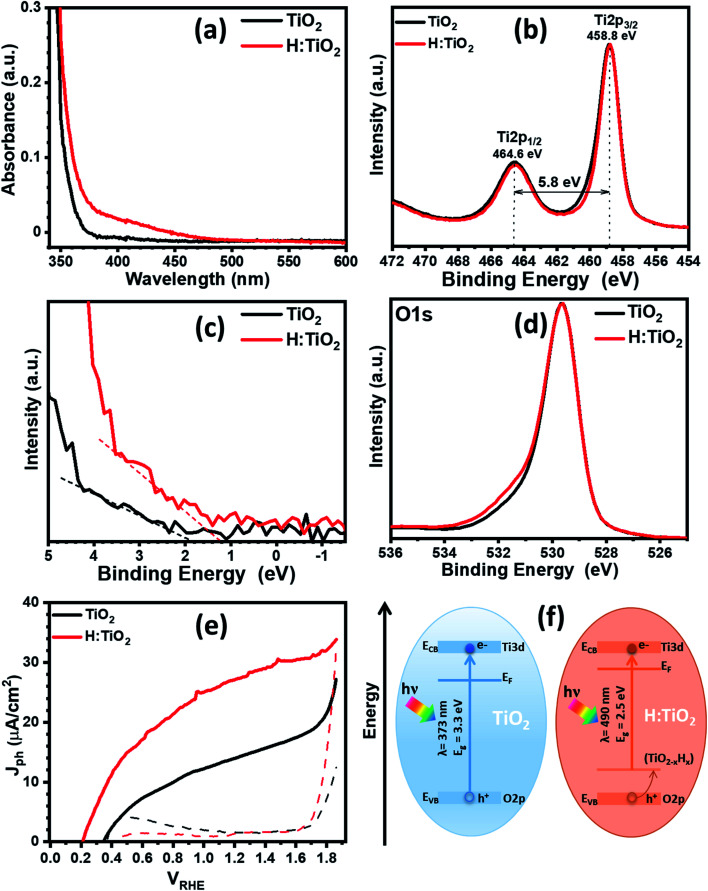
(a) Optical absorption of pristine and hydrogen treated TiO_2_ thin films. (b) Core-level XPS Ti 2p spectra. (c) Valance band spectra of pristine and hydrogen treated TiO_2_. (d) Core-level XPS O 1s spectra. (e) Photocurrent density *versus* applied potential for pristine and hydrogen treated TiO_2_. (f) Band diagram representation of pristine and hydrogen treated TiO_2_. Both representations were built using experimental data from UV-vis spectroscopy and valance band XPS analysis. Conduction band offset was estimated using band gap and valance band offsets.

First, we optimized the temperature for optimized hydrogen doping with respect to the reduction in band gap and upshift of band edge position. Fig. 5a displays the optical absorption spectra of the TiO_2_, H:TiO_2_, samples in the wavelength range 340–600 nm measured by using the integrating sphere. Optical absorption spectra of all the samples, α-Fe_2_O_3_, TiO_2_, H:TiO_2_, Fe_2_O_3_–TiO_2_, and Fe_2_O_3_–H:TiO_2_, in the wavelength range 300–700 nm measured under the same experimental conditions are shown in the ESI (Fig. S10).† In pristine TiO_2_ samples, a steep increase in absorption at wavelengths shorter than ∼373 nm can be attributed to the intrinsic band gap of crystalline TiO_2_. The hydrogen doping of the TiO_2_ sample show a significant shift of absorption edge from higher wavelengths down to 490 nm in the visible light absorption. The calculated values of the band gap energy show that the band gap of the pristine TiO_2_ thin films is approximately 3.30 eV, slightly higher than that of bulk anatase TiO_2_. However, the onset of light absorption in hydrogen doped TiO_2_ thin film is lowered to about 2.5 eV.

To validate the reduction of band gap energy, we have carried out the XPS measurement on both pristine and hydrogen doped TiO_2_ thin films to understand the surface chemical bonds, chemical composition, peak position, and hence the electronic properties. Therefore, the Ti 2p, O 1s, and valance band spectra were measured and analyzed for pristine and hydrogen doped TiO_2_ thin films. The Ti 2p core level XPS spectrum, Fig. 5b, shows that the Ti 2p_3/2_ peak is at 458.8 ± 0.1 eV, which was attributed to Ti^4+^ states for both the samples. However, after hydrogen doping, a slight asymmetric narrowing in the Ti 2p_3/2_ peak can be seen. This narrowing in the peak upon hydrogen doping is likely due to a decrease in Ti^3+^ states, concurrent with the reduction in oxygen content, and to a disorder due to the thermal annealing of TiO_2_ in hydrogen environment.^39^ However, no reduction in the Ti^4+^ was observed in TiO_2_ after hydrogen doping. Fig. 5c shows the valence band spectra obtained for the pristine and hydrogen treated TiO_2_ thin films. The VBM were calculated by linearly extrapolating the peaks to the baselines. The VBM for pristine TiO_2_ thin film was observed at 1.93 eV below the zero potential energy point. However, in the case of hydrogen doped TiO_2_ films, VBM was obtained at 1.27 eV. Therefore, a valence band edge shift by ∼0.66 eV was observed towards the Fermi level. Moreover, small bands occurred above the VBM, and small bands existed close to the binding energy at zero. In light of the first-principles calculations, the up-shift of the VBM is suggested to be the result of defects states above the valance band induced by interstitial hydrogen in the TiO_2_ thin films. Fig. 5d represents the O 1s spectra of pristine and hydrogen doped TiO_2_ samples. The peak at a binding energy of 529.6 ± 0.1 eV corresponds to O–Ti bonds in TiO_2_ in both the samples. However, in hydrogen doped samples, an additional peak can be seen at 531.6 ± 0.1 eV, which is probably due to oxygen vacancies, formation of hydroxyl groups, and Ti–OH bonds.^39,40^ From Fig. S13b and c,† where the XPS spectra of all four samples are shown, it is further visible that Fe_2_O_3_–H:TiO_2_ displays the same narrowing of the Ti^4+^ peak as H:TiO_2_, and a more accentuate OH^−^ peak in the oxygen 1s spectrum, correlating the formation of the iron oxide heterostructure with the generation of oxygen vacancies and/or hydroxyl groups.

The PEC measurement in the form of photocurrent densities *vs.* applied potential (*J*_ph_*vs. V*_RHE_) curves are shown in Fig. 5e for pristine and hydrogen doped TiO_2_ samples under 1 sun illumination. The calculated value of *J*_ph_ for pristine TiO_2_ photoanode was obtained ∼14 μA cm^−2^ at 1.23 V_RHE_. However, hydrogen doping in TiO_2_ enhanced the PEC response significantly and reached a *J*_ph_ to ∼27.4 μA cm^−2^ at 1.23 V_RHE_. It is noticed that hydrogen doping in TiO_2_ thin films greatly enhanced the *J*_ph_ and shifted the photocurrent onset potential to 0.2 V_RHE_. The relatively low value of photocurrent onset potential indicates that the charge separation and transportation in hydrogen doped TiO_2_ thin films are more efficient than the pristine TiO_2_ thin films. We have also calculated the flat-band potential (*V*_fb_) and carrier concentration (*N*_d_) for pristine and hydrogen doped TiO_2_ samples using Mott–Schottky plots. The value of *V*_fb_ was calculated from the intercept of the straight line to the *x*-axis in the linear region of the plot, as shown in Fig. S11.† The pristine TiO_2_ sample exhibit a *V*_fb_ value of 0.29 V_RHE_. However, a shift in *V*_fb_ to 0.15 V_RHE_ was calculated for the H:TiO_2_ photoanode. This significant shift of *V*_fb_ by 0.14 V towards the cathodic side in H:TiO_2_ is likely because the hydrogen doping can enhance the carrier concentration due to increased oxygen vacancies, which could serve as shallow electron donor and upshift the Fermi level towards the conduction band of TiO_2_. Schematic positions of band edges of TiO_2_ and H:TiO_2_ are shown in Fig. 5f. The band alignment of H:TiO_2_ facilitates more efficient charge separation at the semiconductor/electrolyte interface as compared to pristine TiO_2_ by increasing the degree of band bending at the H:TiO_2_ surface. The experimental value of *V*_fb_ for H:TiO_2_ is more cathodic in comparison to the pristine TiO_2_, which favors the water splitting reaction. In summary, the enhancement in photocurrent density in the H:TiO_2_ sample is thanks to the combined effect of decreased bandgap energy, which allows absorption of a wide range of solar radiation, high donor density which is responsible for the shift in Fermi level towards the conduction band, and more negative flat band potential.

**Fig. 6:**
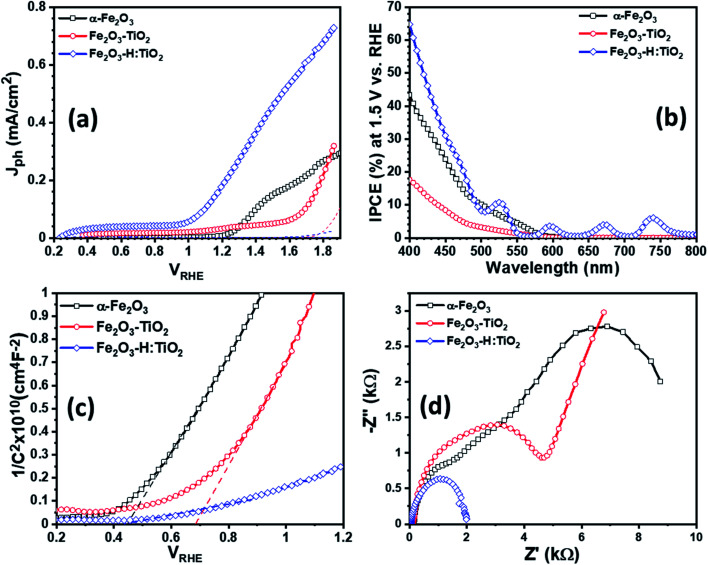
(a) Photocurrent density *versus* applied potential (b) IPCE curves measured at 1.5 V *vs.* RHE for the α-Fe_2_O_3_, Fe_2_O_3_–TiO_2_, and Fe_2_O_3_–H:TiO_2_ heterostructures photoanodes. (c) Mott–Schottky plots for α-Fe_2_O_3_, Fe_2_O_3_–TiO_2_, and Fe_2_O_3_–H:TiO_2_ heterostructures measured in 0.1 M NaOH electrolyte solution under 1 sun illumination. (d) Measured EIS Nyquist plots for α-Fe_2_O_3_, Fe_2_O_3_–TiO_2_ and Fe_2_O_3_–H:TiO_2_ heterostructure.

To examine the PEC performance of the targeted heterostructures photoanodes, Fe_2_O_3_–TiO_2_ and Fe_2_O_3_–H:TiO_2_, the *J*_ph_*vs. V*_RHE_ characteristics under dark and illumination was measured in a three-electrode PEC cell. Fig. 6a depicts the PEC performance of the optimized Fe_2_O_3_–TiO_2_ and Fe_2_O_3_–H:TiO_2_ heterostructures compared with pristine α-Fe_2_O_3_ photoanode. The dark current densities, for the applied potential range up to 1.7 V_RHE_, are negligible for all samples. The *J*_ph_ of the pure α-Fe_2_O_3_ photoanode is ∼10 μA cm^−2^ at 1.23 V *vs.* RHE, which is reasonable as compared to the previously reported values due to the difference in the film thickness and has an onset of photocurrent at 1.15 V_RHE_.^20^ A much lower onset potential of ∼0.35 V_RHE_ and lower *J*_ph_ of ∼35 μA cm^−2^ at 1.23 V_RHE_ obtained for the Fe_2_O_3_–TiO_2_ heterostructure. However, the hydrogen doped TiO_2_ layer in the Fe_2_O_3_–H:TiO_2_ heterostructure substantially shifted the onset of photocurrent to 0.21 V_RHE_ and the *J*_ph_ at 1.23 V_RHE_ increased to 215 μA cm^−2^. The value of *J*_ph_ for Fe_2_O_3_–H:TiO_2_ heterostructure photoanode reached 450 μA cm^−2^ at 1.5 V_RHE_, which is three and nine times the *J*_ph_ for α-Fe_2_O_3_ (153 μA cm^−2^) and Fe_2_O_3_–TiO_2_ (51 μA cm^−2^) photoanodes, respectively. The obtained value of *J*_ph_ for Fe_2_O_3_–H:TiO_2_ heterostructured system is significantly high to that of a pristine α-Fe_2_O_3_ and Fe_2_O_3_–TiO_2_ systems. As the magnitude of *J*_ph_ corresponds to the number of photogenerated holes that reach the electrode surface, the Fe_2_O_3_–TiO_2_ sample exhibits a high recombination of photogenerated electrode and holes at the junction due to the discontinuity of the mismatch of band edge positions, which hinders the water oxidation process at the surface of the Fe_2_O_3_–TiO_2_ photoanode. As we reported in our previous work on hydrogen treated TiO_2_ and predicted in our simulation for this work, hydrogen doping in TiO_2_ can upshift the valance band position and create the lattice defects in TiO_2_. Our simulation results on hydrogen doping in TiO_2_ indicate that heterojunctions of α-Fe_2_O_3_ and hydrogen doped TiO_2_ can lead to considerably enhanced hole transport from α-Fe_2_O_3_ to H:TiO_2_ and, thus, to a more efficient separation of photoexcited electron–hole pairs. Consequently, hydrogen treated Fe_2_O_3_–TiO_2_ photoanode exhibits a higher number of photogenerated holes that can participate in the oxidation process before recombining with excited electrons.

Further, to quantitatively investigate the PEC performance of the photoanodes, incident photon-to-current conversion efficiency (IPCE) measurements were performed at 1.5 V *vs.* RHE in 1.0 M KOH and shown in Fig. 6b. All the samples show photocurrent response to incident light in the wavelength region of 400–580 nm, which matches the bandgap of hematite (∼2.1 eV). The pristine α-Fe_2_O_3_ photoanode exhibits 42% IPCE at the excitation wavelength of 400 nm, which is comparable with the reported IPCE values of α-Fe_2_O_3_ photoanode. A significantly low IPCE, 18%, was recorded in the case of the Fe_2_O_3_–TiO_2_ photoanode. This Fe_2_O_3_–TiO_2_ photoanode also exhibits the low photocurrent density in PEC measurements. However, Fe_2_O_3_–H:TiO_2_ photoanode exhibits substantially high quantum efficiency as compared with pristine α-Fe_2_O_3_ and Fe_2_O_3_–TiO_2_ photoanodes in the wavelength range of 400–550 nm. The IPCE of the Fe_2_O_3_–H:TiO_2_ photoanode reaches to ∼65% at the excitation wavelength of 400 nm, which is 1.5 and 3.6 times that of α-Fe_2_O_3_ (42%) and Fe_2_O_3_–TiO_2_ (18%), respectively.

To gain even more insight into the enhancement of photocurrent density and IPCE values in Fe_2_O_3_–H:TiO_2_ heterostructure photoanodes, Mott–Schottky measurements were performed with applied potential at 1 kHz frequency in the dark. From the Mott–Schottky plots, which represent the changes in capacitance (*C*) against the applied potential, it can be seen that all the samples possess a positive slope (Fig. 6c), which is a characteristic of n-type semiconductors with electrons as majority carriers. The flat band potential (*V*_fb_) and carrier density (*N*_d_) were calculated from the slopes of Mott–Schottky plots. The value of *V*_fb_ was observed to shift towards the more cathodic side from 0.44 V_RHE_ for α-Fe_2_O_3_ to 0.68 V_RHE_ for the Fe_2_O_3_–TiO_2_ sample. However, after the hydrogen treatment of the TiO_2_ layer, the flat band potential remains at the same values to 0.28 V_RHE_ as Fe_2_O_3_–TiO_2_.

Further, the charge transport properties of the Fe_2_O_3_–TiO_2_ and Fe_2_O_3_–H:TiO_2_ photoanodes were investigated by electrochemical impedance spectroscopic (EIS) measurements at 1.23 V_RHE_ applied potential under one sun illumination conditions and compared with α-Fe_2_O_3_. Fig. 6d shows the EIS Nyquist plots for α-Fe_2_O_3_, Fe_2_O_3_–TiO_2_, and Fe_2_O_3_–H:TiO_2_ photoanodes. The series resistance (*R*_s_) at the interface between the ITO substrate and α-Fe_2_O_3_ and Fe_2_O_3_–TiO_2_ layers display a substantial reduction from *ca.* 140 Ω cm^−2^ for the pristine α-Fe_2_O_3_ sample, to 120 Ω cm^−2^ for Fe_2_O_3_–TiO_2_, to *ca.* 40 Ω cm^−2^ for the Fe_2_O_3_–H:TiO_2_ photoanode surface. The radii of the semicircle in the EIS plots from the Fe_2_O_3_–H:TiO_2_ heterostructure is much smaller than that from Fe_2_O_3_–TiO_2_, which indicates that the hydrogen treatment of the TiO_2_ layer changes the charge distribution in Fe_2_O_3_–H:TiO_2_. The new charge distribution results in a lower magnitude of the equivalent series resistance in Fe_2_O_3_–H:TiO_2_ heterojunction photoanodes indicating strongly improved charge transport properties as compared to Fe_2_O_3_–H:TiO_2_.

Finally, in order to assess the durability of the photoanodes, chronoamperometry was performed on the Fe_2_O_3_–TiO_2_ and the Fe_2_O_3_–H:TiO_2_ electrodes (as presented in Fig. S12 in the ESI†): the resulting photocurrent density, under a bias voltage of 0.5 V_RHE_ and an illumination of 1 sun, was found to be stable around a value of 0.4 mA cm^−2^ for up to 500 minutes for Fe_2_O_3_–H:TiO_2_, with no sign of decreasing, while the non-treated sample displayed a lower photocurrent (0.23 mA cm^−2^) at the start, which halved after less than 300 minutes of continuous use. This sustained stability of Fe_2_O_3_–H:TiO_2_ electrodes confirms the applicability of the device.

## 4. Conclusion

First-principles calculations predict that hydrogen treatment induce states above the valance band, which can transfer holes through the heterostructure junction. The predictions are validated with experimental observation obtained on sol–gel grown TiO_2_ thin films. Optical absorption, XPS analysis, and (photo)electrochemical measurements show that the tailoring in optical band gap, shift in valence band position, and change in electrical properties in hydrogen doped TiO_2_ results in a type II band alignment in the Fe_2_O_3_–H:TiO_2_ heterostructure. The Fe_2_O_3_–H:TiO_2_ heterostructure reduces the electron–hole recombination sharply at the junction and improves the water oxidation performance. This study shows that hydrogen treatment can enhance the photoelectrochemical response of the Fe_2_O_3_–TiO_2_ heterostructure, thanks to the formation of type II band alignment at the interface junction. The theoretical and experimental strategies can be applied to other oxides based heterostructures and might become important tools for engineering efficient and stable photoelectrodes.

## Author contributions

A. P. S., B. W. and A. H. conceived the idea and designed the experimental work. A. H. and R. B. W. supported the work through theoretical simulations. A. P. S. have prepared and characterized the samples for experimental work and R. B. W. preformed the thermotical simulation. A. P. S., B. W. and A. H. analyzed the experimental data for the manuscript. The manuscript was written through the contributions of all authors. All authors have given approval to the final version of the manuscript.

## Conflicts of interest

There are no conflicts to declare.

## Supplementary Material

RA-011-D0RA09655E-s001
