# Supplementary material for: Hydrogen induced interface engineering in Fe2O3–TiO2 heterostructures for efficient charge separation for solar-driven water oxidation in photoelectrochemical cells
Source: RSC Adv. 2021 Jan 21;11(8):4297–307. doi: 10.1039/d0ra09655e (PMC8694353; doi:10.1039/d0ra09655e)
Supplement: RA-011-D0RA09655E-s001 [file RA-011-D0RA09655E-s001.pdf]

Electronic Supplementary Material (ESI) for RSC Advances.
This journal is © The Royal Society of Chemistry 2021

Supplementary Information

Hydrogen Induced Interface Engineering in Fe_2O_3-TiO_2 Heterostructures for Efficient Charge Separation for Solar-driven Water Oxidation in Photoelectrochemical Cells

Aadesh P. Singh[1,*], Richard Baochang Wang[2], Camilla Tossi[1], Ilkka Tittonen[1], Björn Wickman[2], Anders Hellman[2,*]

[1]Department of Electronics and Nanoengineering, School of Electrical Engineering, Aalto University, P.O. Box 13500, 00076 AALTO, Finland

[2]Division of Chemical Physics, Department of Physics, Chalmers University of Technology, SE-412 96 Göteborg, Sweden

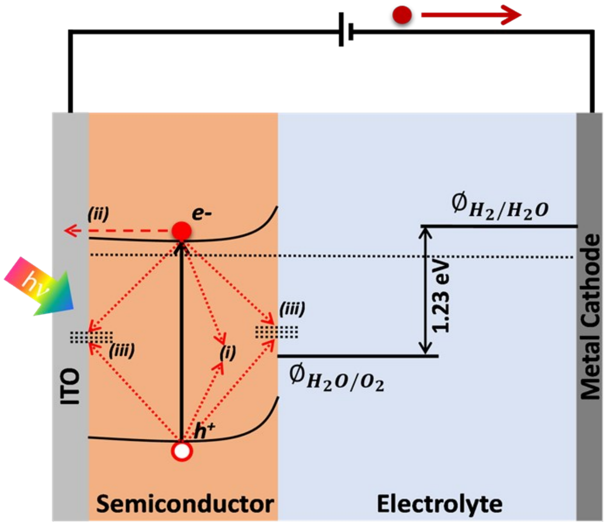

Figure S1: Schematic band alignment of hematite photoanode for PEC water oxidation. Creation of photogenerated electron-hole pair, migration of holes to the hematite surface for water oxidation to oxygen, and migration of electrons to the back charge collector metal cathode for water reduction to hydrogen. The four possible recombination sites that are unfavorable to complete the water oxidation and reduction in a PEC cell are: i) bulk charge recombination, ii) poor charge collection efficiency, iii) surface trapping and recombination and iv) interfacial carrier trapping and recombination.

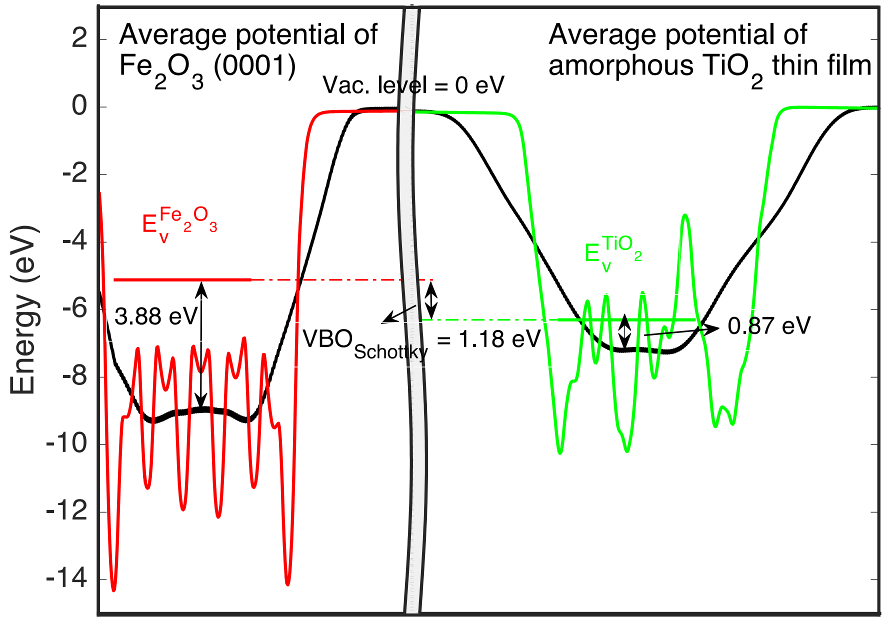

Figure S2: The calculated the VBO of individual systems of Fe_2O_3 (0001) and a-TiO_2 thin film.

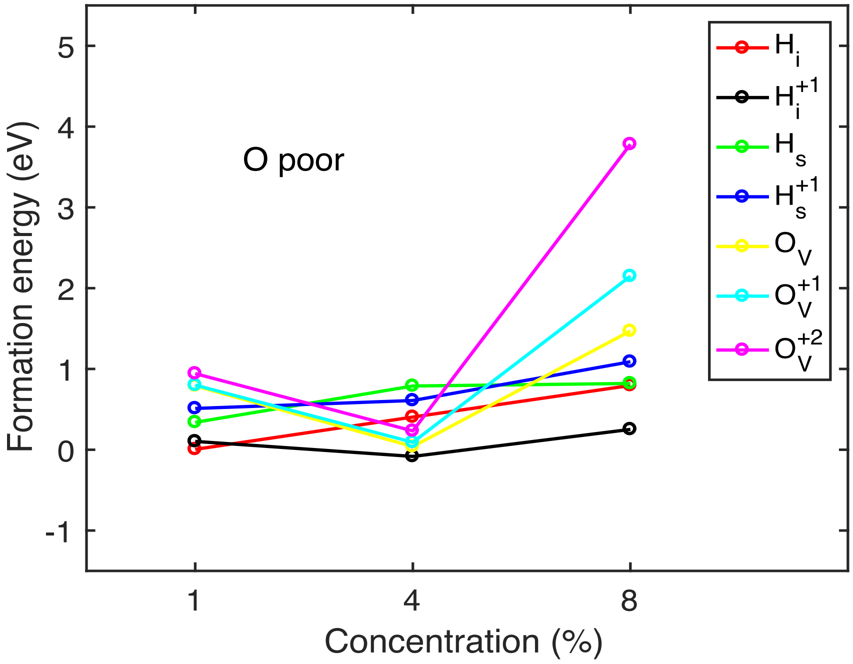

Figure S3: The calculated formation energy of various defects in anatase varying with the defect concentration under O-poor conditions. The Fermi energy is set to $E_f = 3.0$ eV

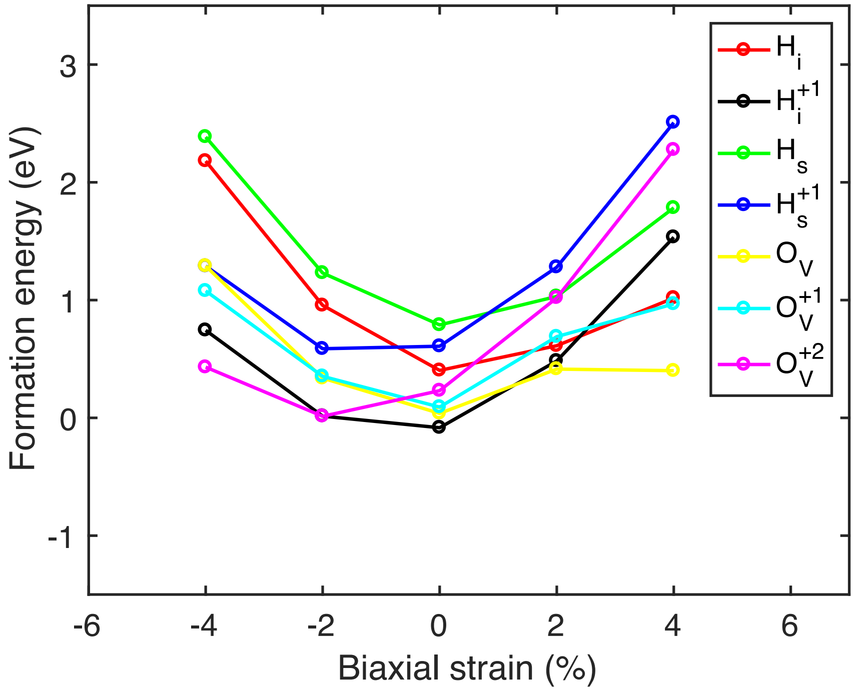

Figure S4: The calculated formation energy of different charged/neutral defects under biaxial strain from -4% to +4% under O-poor conditions. The Fermi energy is set to $E_f = 3.0$ eV.

Table S1. Formation energy of H interstitial, substitutional H and O vacancy in a-TiO_2 calculated with the PBE + U method.

H in a-TiO_2	fromation energy
**H_i_2O**	8.77
**H_i_3O**	-0.407
**H_i_1x2**	-1.04
**H_i_1x3**	-1.69
**H_s**	3.838
**O_v**	7.840

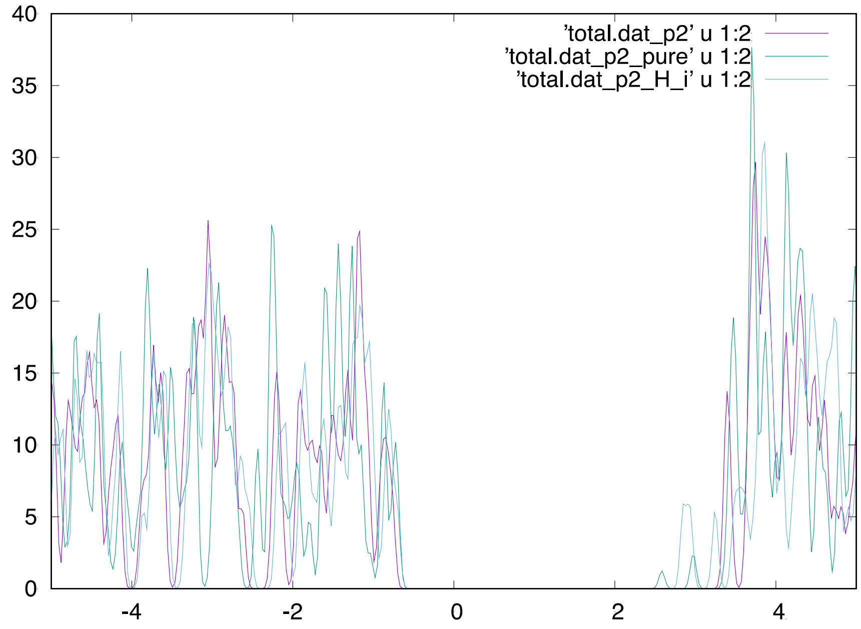

Figure S5: The DOS of pure anatase, H_{i} and H_{i}^{+} doped anatase under 2% tensile biaxial strain.

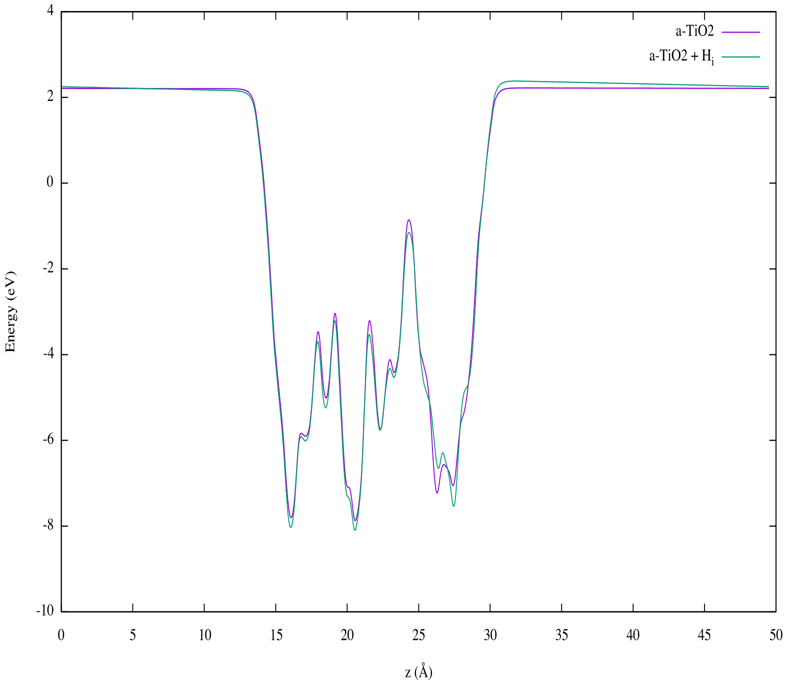

Figure S6: The calculated electrostatic potential of the H doped a-TiO_2 as compared with pristine a-TiO_2.

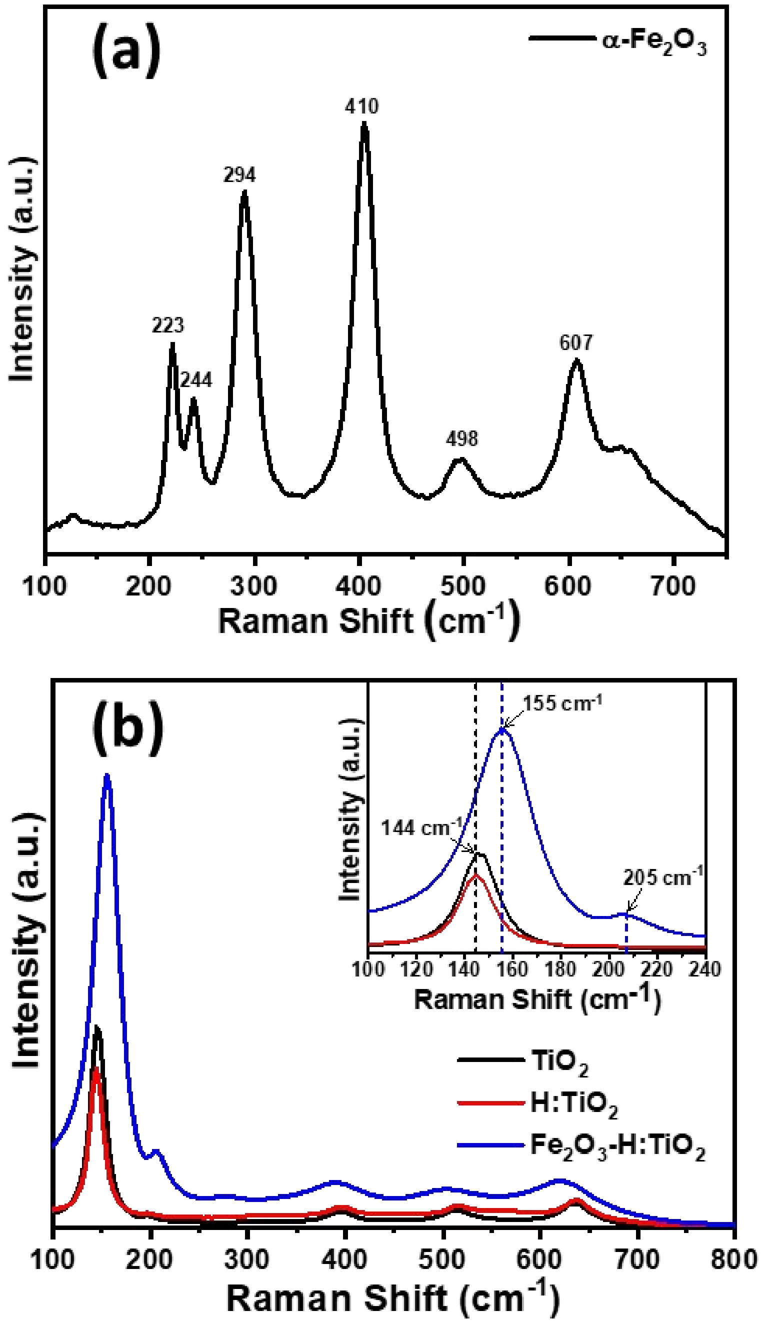

Figure S7. (a) Raman spectra of α-Fe_2O_3, and (b) Raman spectra of TiO_2, H:TiO_2 and Fe_2O_3-TiO_2 heterostructure thin films recorded from wavenumbers in the range of 100-900 cm^{-1}, Inset of (b) shows the no shift in Eg mode of TiO_2 upon hydrogen doping, however an up-shift of Eg mode of TiO_2 from 141 to 155 cm^{-1} can be seen in Fe_2O_3-TiO_2 heterostructure.

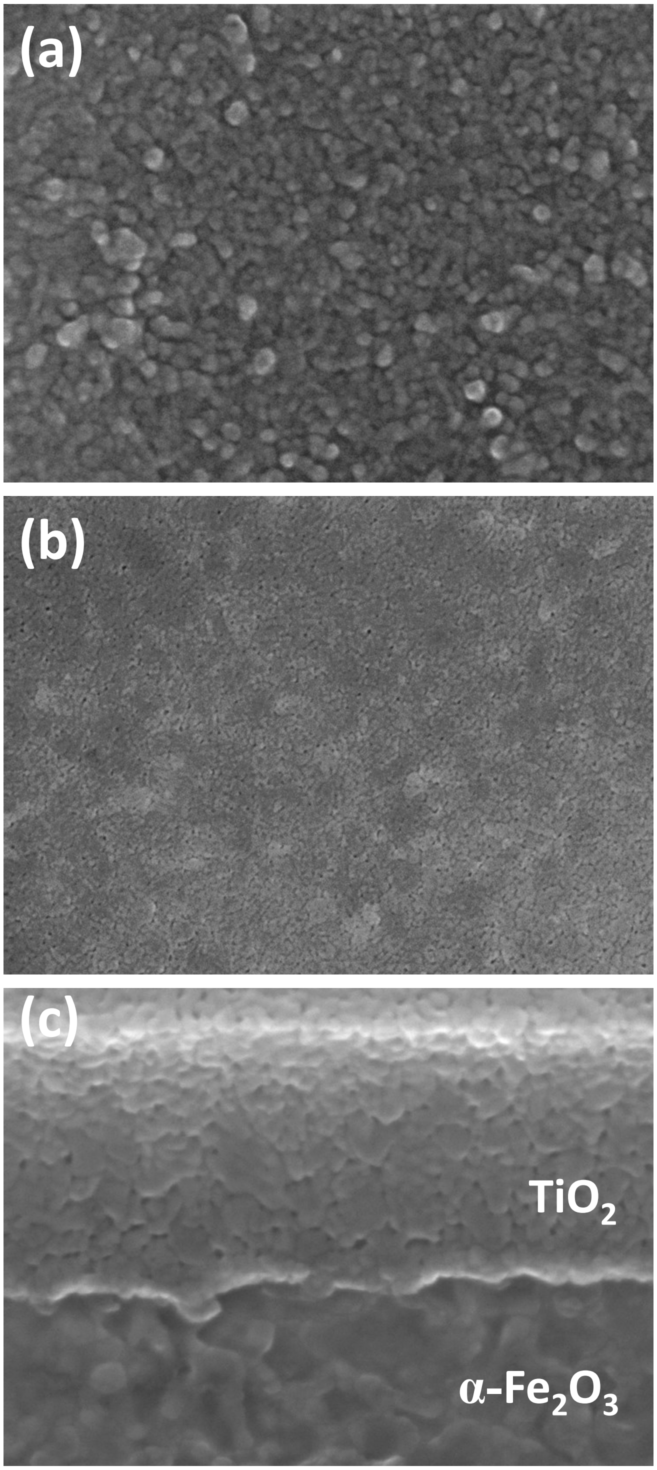

Figure S8: SEM images of pristine α-Fe_2O_3 and (b) pristine TiO_2 and (c) Fe_2O_3-TiO_2 heterostructures annealed at 350 ^{o}C.

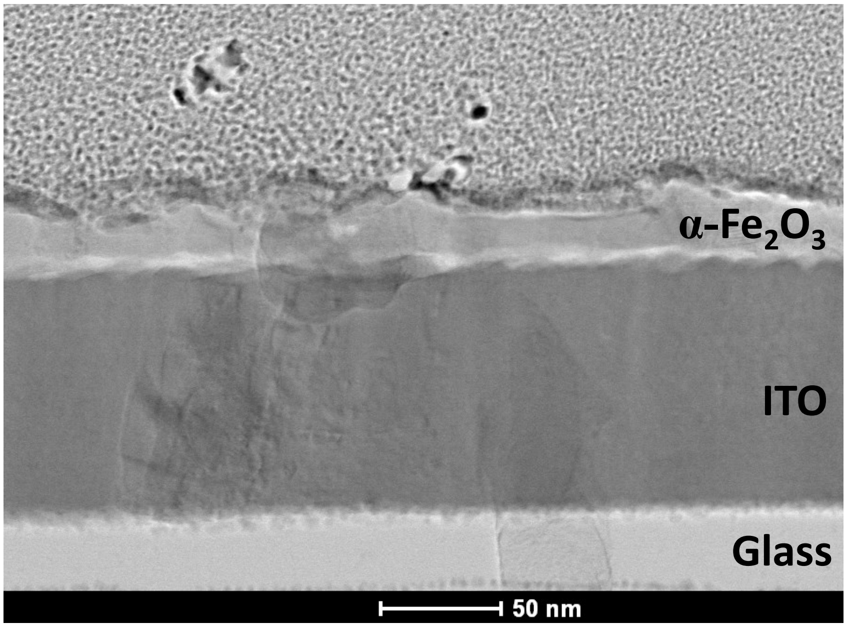

Figure S9: TEM images of pristine α-Fe_2O_3 annealed at 350 °C.

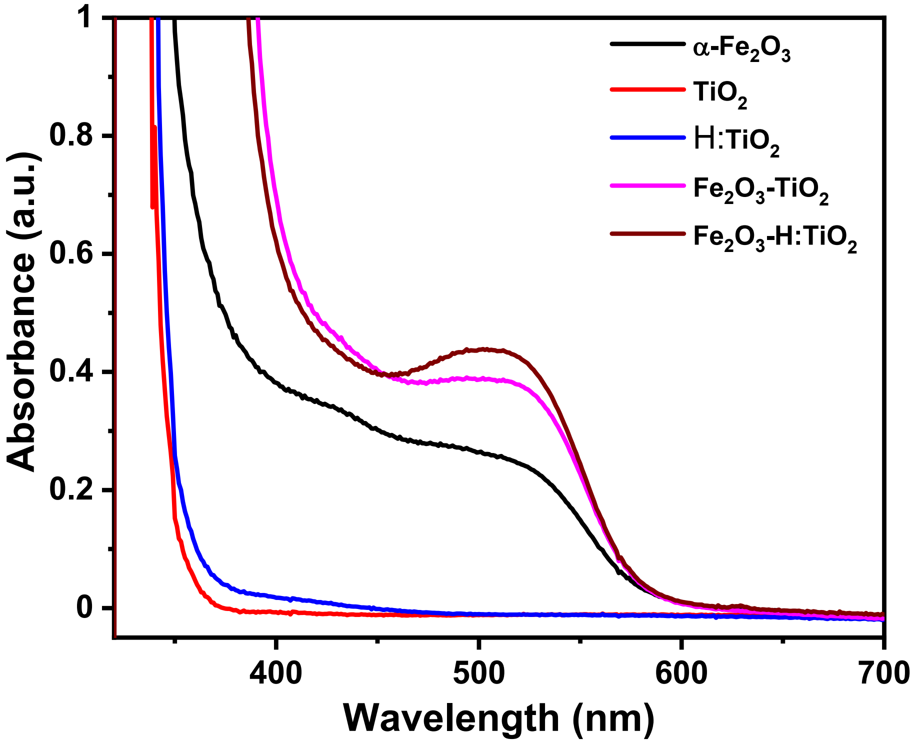

Figure S10: Optical absorption spectra of TiO_2, H:TiO_2, α-Fe_2O_3, Fe_2O_3-TiO_2, and Fe_2O_3-H:TiO_2 heterostructures.

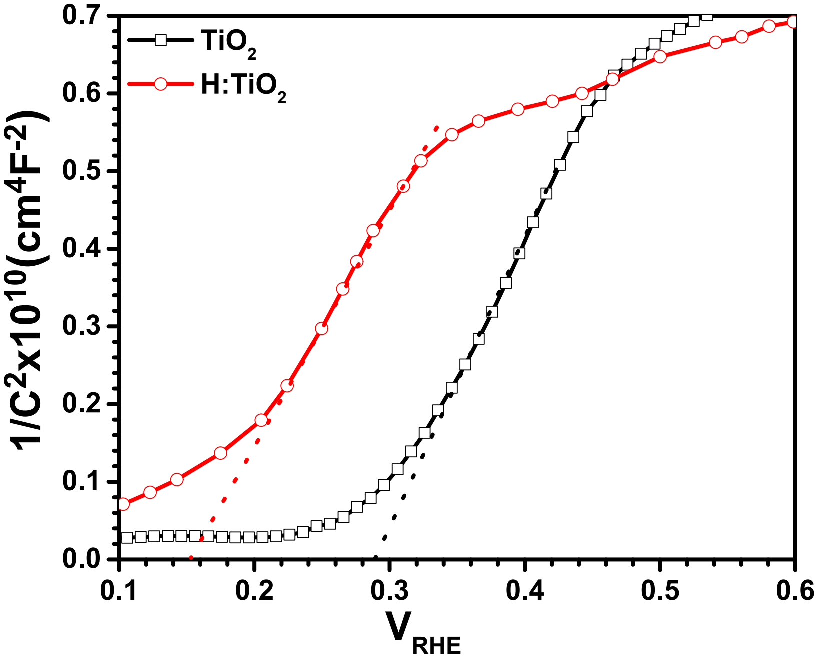

Figure S11: Mott-Schottky curves measured in dark condition for pristine and hydrogen doped TiO_2 photoanodes obtained at 1 kHz.

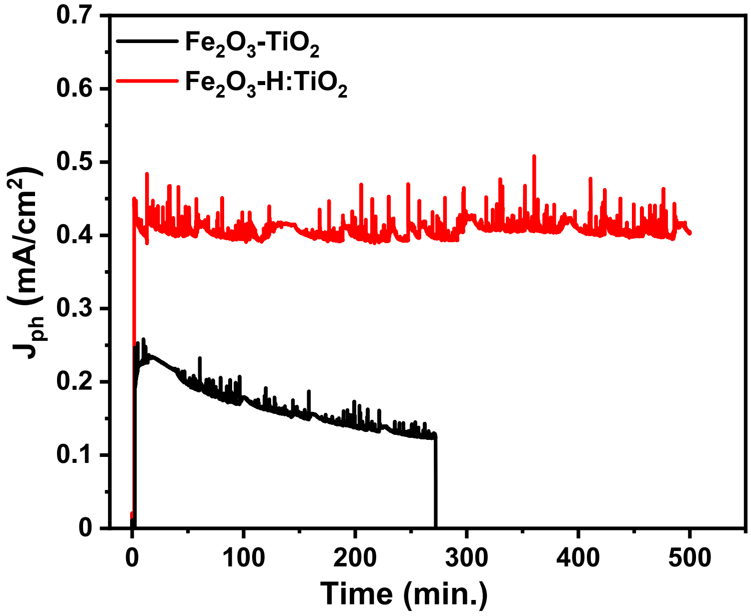

Figure S12: Long-term photoanode stability test of Fe_2O_3-TiO_2 and Fe_2O_3-H:TiO_2 heterostructure under 1 Sun illumination. In order to check the stability of the Fe_2O_3-H:TiO_2 heterostructure photoelectrodes, chronoamperometric studies were performed. A bias potential of 0.5 V_{RHE} in 0.1 M NaOH solution was applied to the photoelectrode and the photocurrent density is measured for 600 minutes. A stable photocurrent density was found. It is worth noting that the nominal decrease in photocurrent densities in this stability test measurements are achieved at 0.5 V_{RHE} applied biases.

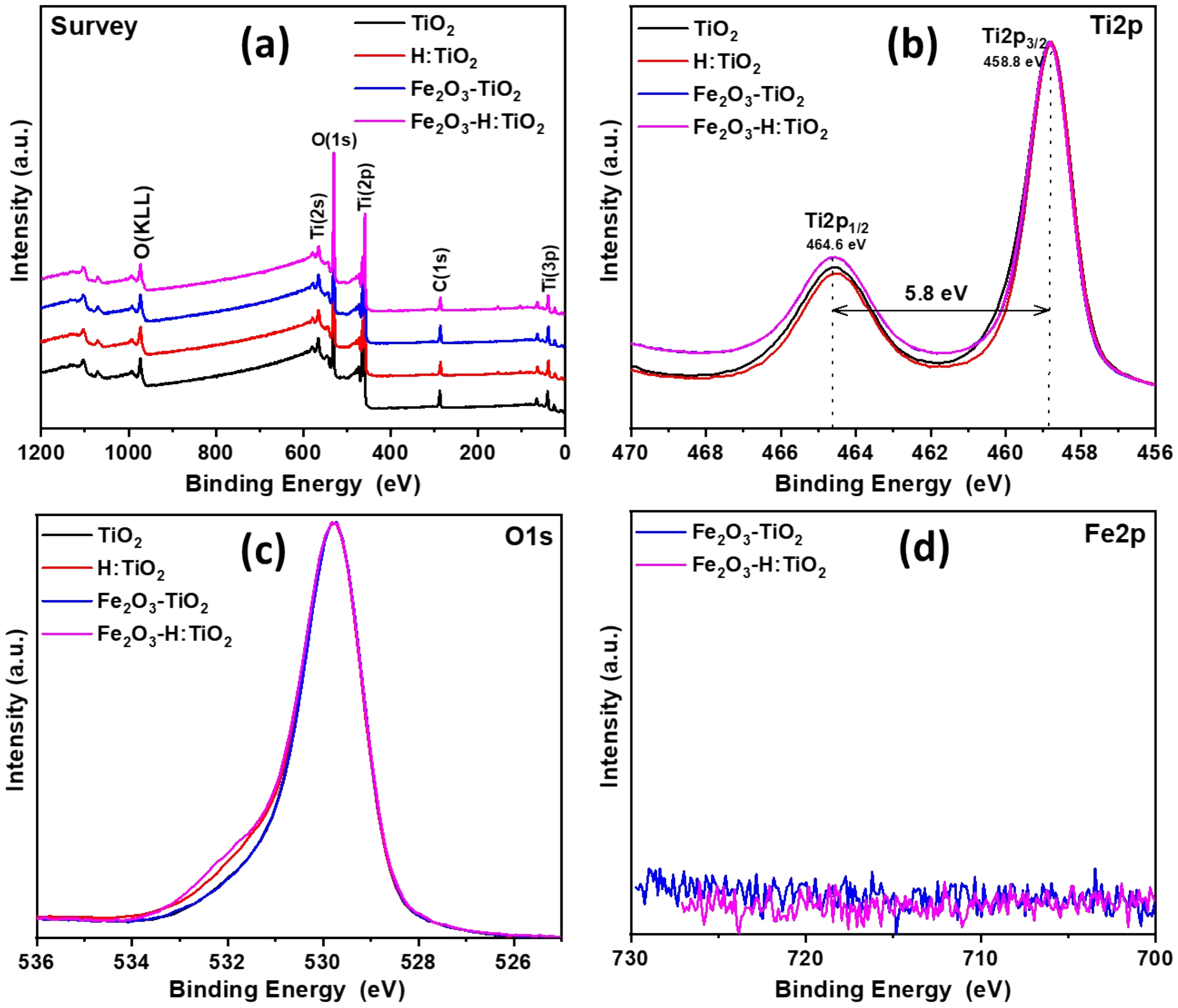

Figure S13: (a) XPS survey spectra (b) Ti2p core-level (c) O1s core-level spectra for the pristine TiO_2, H:TiO_2, Fe_2O_3-TiO_2, and Fe_2O_3-H:TiO_2 specimens and (d) Fe2p core-level spectra for Fe_2O_3-TiO_2, and Fe_2O_3-H:TiO_2 specimens.
